# Creativity in Music: The Brain Dynamics of Jazz Improvisation

**DOI:** 10.1111/nyas.70042

**Published:** 2025-09-27

**Authors:** Patricia Alves Da Mota, Henrique Miguel Fernandes, Ana Teresa Lourenço Queiroga, Eloise Stark, Jakub Vohryzek, Joana Cabral, Ole Adrian Heggli, Nuno Sousa, Gustavo Deco, Morten Kringelbach, Peter Vuust

**Affiliations:** ^1^ Center For Music in the Brain (MIB), Department of Clinical Medicine Aarhus University/The Royal Academy of Music Aarhus Denmark; ^2^ Centre For Eudaimonia and Human Flourishing, Linacre College University of Oxford Oxford UK; ^3^ International Centre for Flourishing Universities of Oxford, Aarhus, and Pompeu Fabra Oxford UK; ^4^ Center For Brain and Cognition, Computational Neuroscience Group Universitat Pompeu Fabra Barcelona Spain; ^5^ Life and Health Sciences Research Institute (ICVS), School of Medicine University of Minho Braga Portugal; ^6^ Institució Catalana de La Recerca i Estudis Avançats (ICREA) Barcelona Spain

**Keywords:** creative behavior, fMRI, jazz, Leading Eigenvector Dynamical Analysis, LEiDA, music improvisation

## Abstract

Jazz improvisation is a controlled yet ecologically valid framework for investigating spontaneous creative behavior. We examined spatiotemporal brain dynamics when skilled musicians applied different strategies to improvise on a jazz standard. We performed rest and task‐based functional magnetic resonance imaging on 16 skilled jazz pianists playing “Days of Wine and Roses”, with varying levels of improvisation freedom: (1) playing the melody from memory (*byHeart*); (2) improvising on the melody (*iMelody*); and (3) freely improvising (*iFreely*) on the chord changes. Behaviorally, higher levels of improvisational freedom were associated with a larger number of notes, greater melodic entropy, and reduced pitch predictability. Using the Leading Eigenvector Dynamics Analysis (LEiDA), we found increased activity in the reward system for all conditions compared to rest, including the orbito‐frontal cortex. In the improvisation conditions compared to rest, there was a significantly higher probability of a brain state comprising auditory and sensorimotor areas related to musical performance and right insula belonging to the posterior salience network. The highest level of improvisational freedom (*iFreely*) had a higher occurrence of a brain substate, including the default mode, executive control, and language networks. These networks are involved in planning complex behaviors, decision‐making, and motor control—all relevant for understanding neural signatures of creativity.

## Graphical abstract

Using rest and task‐based fMRI, we studied brain dynamics in 16 jazz pianists under three conditions: playing a melody from memory (*byHeart*), improvising on melody (*iMelody*), and freely improvising on chord changes (*iFreely*). Increased improvisational freedom revealed greater note quantity and melodic entropy, and reduced pitch predictability. Neural activity engaged reward, auditory, sensorimotor, and salience networks, with *iFreely* activating default mode, executive control, and language networks critical for spontaneous creativity.

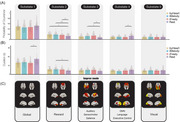

## 1 Introduction

Creativity is a complex and multifaceted phenomenon, commonly defined as the capacity to generate ideas or products that are both novel and appropriate to a given context [1]. One influential theoretical view suggests that creativity emerges from the interplay between constraint and freedom, or between predictability and surprise, where the familiar is recombined in original and unexpected ways [2]. Despite decades of research, studying creativity in action remains a challenge due to its abstract nature and the difficulty of capturing real‐time generative processes.

Musical improvisation provides a valuable window into human creativity. It is spontaneous yet bounded by stylistic and structural constraints and requires continuous interaction between perception, planning, execution, and evaluation [3, 4]. Furthermore, there is an observable outcome, which can be evaluated both with qualitative and quantitative measures. As such, it serves as an ecologically valid model for understanding the neural mechanisms underlying musical creativity [5, 6, 7, 8, 9, 10, 11].

Jazz improvisation involves the real‐time generation of the melodic, harmonic, and rhythmic material within a structured musical context. It requires musicians to communicate while simultaneously expressing and anticipating spontaneous musical ideas [12]. Here, one of the most important purposes of compositions is to serve as a melodic, harmonic, and rhythmic framework for soloists. In the present study, we aimed to study two important modes of improvisation, with different levels of improvisational freedom (soloing on the melody and freely on the chords), with a novel analysis approach—the Leading Eigenvector Dynamics Analysis (LEiDA)—to reveal the underlying brain dynamics over time. This allows for studying the dynamical change in brain processing during conditions.

The creative process in jazz improvisation can be broken down into different strategies used by jazz musicians. One common strategy is to use the melody as the starting point for the improvisation [13]. The outcome often becomes hummable and as such could be related to the emotional processing known to be associated with the perception of songs. Many jazz musicians who use this approach are known to accompany their instrumental improvisation with vocalizations. Another strategy is to improvise more freely but still be constrained by the chord scheme belonging to a specific tune [14, 15]. Here, jazz musicians use their skills and previously practiced melodic and harmonic material as building blocks for creating musical pieces that are novel and engaging [13, 16]. In real‐world jazz improvisation, these two strategies for improvisation are typically used interchangeably, where there is a seamless transition between parts that refer mostly to the melody or to the chord changes.

Neuroimaging studies—primarily using functional magnetic resonance imaging (fMRI), electroencephalography, and positron emission tomography—involving jazz musicians performing in situ or under ecologically valid task designs, have consistently implicated both domain‐specific and domain‐general brain networks. These include regions related to motor sequence planning and execution, attentional control, voluntary action selection, sensorimotor integration, emotional processing, and interpersonal coordination [4, 17, 18]. These networks also include prominent roles of prefrontal brain regions, such as the presupplementary motor area, medial prefrontal cortex, inferior frontal gyrus (IFG), dorsolateral prefrontal cortex, dorsal premotor cortex, and auditory cortices [14, 19]. This distributed activation reflects the multifaceted demands of improvisation, which requires musicians to simultaneously perform, monitor, and adapt their playing in response to the surrounding musical context [16]. Underlying these processes are predictive mechanisms that support the generation and evaluation of forthcoming musical events, enabling musicians to anticipate harmonic progressions, synchronize with coperformers, and plan motor actions accordingly. Such predictive functions are thought to engage domain‐general processes like forward motor modeling and expectancy generation, as well as domain‐specific mechanisms for auditory sequence prediction [20].

Only a few studies have investigated musical improvisation from a whole‐brain connectivity perspective and point to a large repertoire of brain states involving functional brain connectivity among frontal and parietal regions within default mode, salience, and executive brain networks [7, 19, 21, 22]. These networks are similar to those found in more general creativity tasks, such as when participants perform the classic divergent thinking tasks, and indicate that the creative process in improvisation mirrors broader creativity processes found in cognitive tasks [23, 24].

Recent state‐of‐the‐art neuroimaging methods, including dynamic functional connectivity measures and time‐resolved network analysis, have made it possible to investigate the changing predictive networks and brain states underlying different experimental conditions in real time [25, 26, 27]. By using methods such as the LEiDA, it is possible to track brain state transitions during different experimental conditions. This allows for observation of the rapidly changing brain networks that are particularly characteristic of the real‐time dynamics of musical improvisation.

In the present study, we asked professional pianists to either improvise on the melody or improvise more freely within the harmonic constraints of the chord changes (*iMelody* and *iFreely*, respectively) and used a novel analysis framework revealing the underlying spatiotemporal brain dynamics. This allowed for (1) determining the brain dynamics involved in these different strategies used for musical improvisation, and (2) identifying the local neural drivers supporting the specific transitions of brain dynamics between different levels of improvisational freedom, with *iMelody* being more constraint than *iFreely*. Using the LEiDA approach, we examined how these different strategies were accompanied by spatiotemporal dynamics over time (quantified as probabilistic metastable substates), and how this can lead to different dynamic brain processing depending on the level of freedom in the improvisational process.

Analysis of the statistical properties of the music produced by the jazz musicians showed that the levels of improvisational freedom were indeed reflected in the music. At the brain level, higher levels of improvisations (*iMelody* and *iFreely*) corresponded to significantly higher probability of occurrence (POc) of brain networks predominantly comprising auditory, sensorimotor, and posterior salience networks (SNs). Additionally, when the highest level of freely improvising (i.e., *iFreely*) was characterized by a higher occurrence of a specific brain substate including default mode network (DMN), executive control network (ECN), and language network.

## 2 Materials and Methods

### 2.1 Participants

The total sample consisted of 24 right‐handed male musicians with normal hearing and no history of neurological disease. Eight participants were excluded from the analyses: two found out that they were claustrophobic, and six were excluded due to excessive head movement. Our final sample resulted in 16 participants (mean age 28.0 ± 8.7 years). All participants were proficient in jazz piano playing (with at least 5 years of jazz‐specific experience). On average, participants had 16.6 ± 9.6 years of overall piano playing experience and 10.9 ± 7.1 years of dedicated jazz practice. They reported practicing an average of 1.9 ± 0.9 h per day, and 22 ± 7.7 days of practicing per month, and had jazz as their main style of music. They were all students at the Royal Academy of Music or played semi‐professionally or professionally. All participants gave written consent to participate in the study. The study was approved by the local ethics committee, and it was undertaken in accordance with the Helsinki Declaration. It should be noted that all participants were male, which may limit the generalizability of the findings.

### 2.2 Stimuli and Procedure

We acquired fMRI while participants were playing on an MRI‐compatible keyboard in four different conditions in a predefined randomized order while listening to a backing track composed of chords, bass, and rhythm of the jazz standard “Days of Wine and Roses” (DWR). Participants were asked to:

- play the melody of DWR from memory (*byHeart*)
- play from a score sheet (*Read*), which featured an alternative melody composed specifically for this experiment on the chord scheme of DWR
- improvise on the melody (*iMelody*), that is, play melodically as if they were to create a new melody for the chord scheme of DWR
- improvise freely on the chord scheme for DWR (*iFreely*).

**FIGURE 1:**
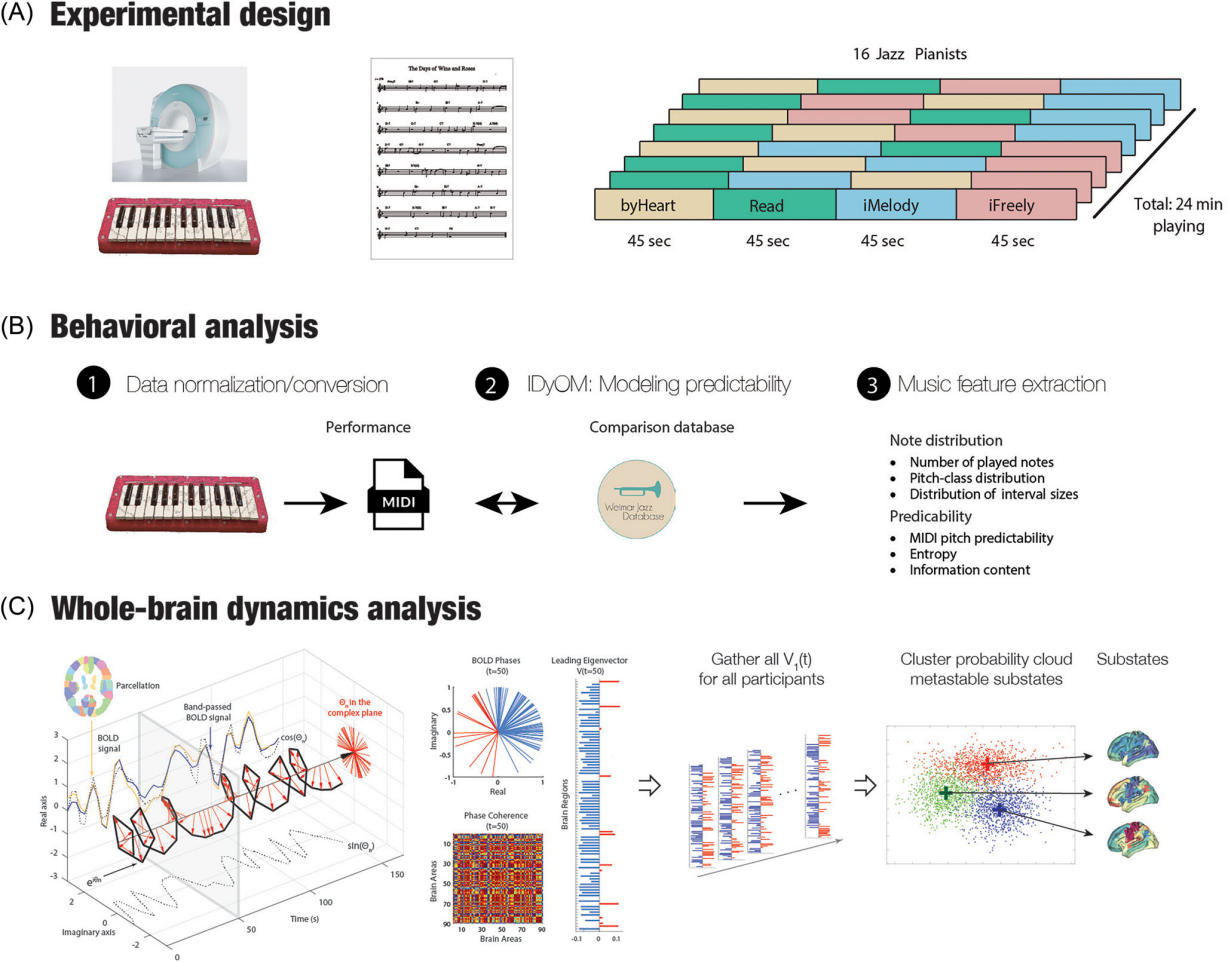
Creativity in music: Experimental protocol and methods. (A) Professional pianists improvised over the jazz standard “Days of Wine and Roses” inside of the MRI scanner using a 25 keys MRI‐compatible keyboard. There were four different conditions: (1) play from memory (*byHeart*); (2) play from a score sheet (Read); (3) improvise by melody (*iMelody*); and (4) freely improvise (*iFreely*). (B) The improvisations were analyzed using a process modeling the pitch predictability by generating a model of probabilistic predictions using Information Dynamics of Music (IDyOM). This allowed for music feature extraction of note distribution and predictability, specifically the number of played notes, pitch‐class distribution, and distribution of interval sizes as well as MIDI pitch predictability, entropy, and information content. (C) We analyzed the brain signals by fingerprinting the whole‐brain dynamics of the music paradigm. Abbreviation: BOLD, blood oxygenation level‐dependent.

Each of the remaining three conditions lasted for 45 s (one time through the chord progression) and was repeated eight times, resulting in a total of 18 min of usable data per participant (6 min per condition) (Figure 1A). There were no visual cues or scores used in conditions a, c, and d.

To ensure that there were no image artifacts, we used a custom‐made MRI‐compatible fiber optic piano keyboard [28]. The keyboard, consisting of 25 full‐size keys, covered two full octaves, and its lightweight and slim design allowed it to be positioned on the participants’ laps, such that all keys could be reached by moving only the forearm. Participants were instructed to only play monophonically with their right hand. Output from the keyboard was interpreted into a Musical Instrument Digital Interface (MIDI) signal by a microcontroller outside of the scanner room. Piano sounds were generated by a Roland JV‐1010 hardware synthesizer based on this MIDI signal. The piano sound from the synthesizer was subsequently mixed together with a backing track and delivered to the participants through OptoACTIVE noise‐cancelling headphones. The backing track was prepared using “Band‐in‐a‐box.” It consisted of bass and drums in a traditional jazz swing style, playing through the changes of DWR (tempo: 84 beats per minute on the half note).

The instructions for each condition were controlled by a PsychoPy [29] script on a laptop computer. An MRI‐compatible screen was used to project the instructions, and participants viewed it using a mirror that was attached to the head coil. Participants were informed of the conditions before going inside the scanner, and they were allowed to play two times the score sheet outside the scanner, to make sure they would understand that they needed to read from a score inside the MR scanner. Inside the scanner, participants received the information through the screen about which condition they should play.

### 2.3 Image Acquisition and Processing

All participants underwent the same imaging protocol using a 32‐channel head coil in a Siemens 3T Trim Trio magnetic resonance scanner located at Aarhus University Hospital, Denmark. Whole‐brain T1‐weighted and task‐based fMRI images were acquired for each participant.

### 2.4 Anatomical Scan Acquisition

The 3D T1‐weighted sequence was performed with the following parameters: sagittal orientation; 256 × 256 reconstructed matrix; 176 slices; slice thickness of 1 mm; echo time (TE) of 3.7 ms; repetition time (TR) of 2420 ms; flip‐angle (a) of 9.

### 2.5 fMRI Acquisition

A multi‐echo EPI‐sequence was acquired with a total of 1484 volumes and with the following parameters: voxel size of 252 × 252 × 250 mm; 54 slices; slice thickness of 2.50 mm; multi‐echo time: TE1 = 12 ms, TE2 = 27.52 ms, TE3 = 43.04 ms, TE4 = 58.56 ms; repetition time (TR) of 1460 ms; flip‐angle (a) of 71. Only the second echo was used in our analysis.

### 2.6 Processing of Behavioral Data

A comprehensive feature analysis was conducted on the collected music data, comprising a total of 384 music files (16 participants × 3 conditions × 8 trials), and was structured into three main steps.

First, raw music data in a three‐column format (note onset in seconds, MIDI pitch, and velocity for each key press event) were cleaned and converted to standard MIDI format using in‐house MATLAB scripts. Due to limitations of the MRI‐compatible keyboard, velocity could not be reliably captured and was thus reduced to binary‐like values (0.787402 for pressed notes, 0 for releases). To ensure a monophonic structure compatible with Information Dynamics of Music (IDyOM) used in a subsequent stage of the analysis, MIDI files were structured to follow a strict “pressed note – released note” sequence. When this structure was violated, correction rules were applied. Files beginning with released notes, an artifact of participants pressing keys before the actual recording started, those notes were deleted. For consecutive pressed notes without interleaved releases, an artificial release with the same MIDI pitch as the preceding pressed note was inserted. In rare cases of simultaneous note presses, only the highest‐pitched note was retained to preserve the melodic line.

Second, we modeled pitch predictability and melodic entropy using IDyOM v1.6 [30], trained on a curated subset of the open‐source Weimar Jazz Database (WJazzD) [31]. WJazzD includes a range of jazz styles (bebop, traditional, free jazz) and features solos performed on various monophonic instruments. The original database contained 456 files with metadata, but we excluded those lacking clear key signatures or adequate jazz standard representation. While some solos labeled as free jazz involve tonal freedom (which the *iFreely* condition does not), they still fit within a data‐driven framework of the database. Many of WJazzD solos featured modulations or used modes/chromatic structures unsupported by IDyOM's major/minor key system. Two expert annotations were added where necessary and resulted in 431 MIDI files used for IDyOM's model. After running IDyOM's automatic viewpoint selection, the final model used cpitch and cpintfre as source viewpoints, with cpitch as the target.

Third, musical features such as the number of played notes, pitch‐class distribution, and interval distribution were extracted using the MIDI Toolbox [32]. Comparisons across playing conditions were visualized using boxplots (Seaborn with open‐source *statannot* Python package), and statistical significance was assessed with Wilcoxon tests using a significance level of α = 0.05.

### 2.7 Processing of Neuroimaging Data

The fMRI data were processed using MELODIC (Multivariate Exploratory Linear Decomposition into Independent Components) [33], part of FSL (FMRIB's Software Library, www.fmri.ox.ac.uk/fsl). The default parameters of this imaging preprocessing pipeline were used for all of the 16 participants: motion correction using MCFLIRT [34]; nonbrain removal using Brain Extraction Tool [35]; spatial smoothing using a Gaussian kernel of full width at half maximum 5 mm; grand‐mean intensity normalization of the entire 4D dataset by a single multiplicative factor; and high‐pass temporal filtering (Gaussian‐weighted least‐squares straight line fitting with sigma = 50 s). FSL tools were used to extract and average the time courses from all voxels within each cluster in the AAL‐90 atlas [36].

### 2.8 Dynamic Functional Connectivity Analysis

We applied a data‐driven approach to capture BOLD phase‐locking (PL) patterns from fMRI data at a single TR resolution with reduced dimensionality (i.e., the LEiDA) [37]. First, the BOLD signals in the *N* = 90 brain areas were band‐pass filtered between 0.02 and 0.1 Hz, and subsequently, the phase of the filtered BOLD signals was estimated using the Hilbert transform [37, 38]. The Hilbert transform expresses a given signal *x* as *x*(*t*) = *A*(*t*)*cos(*q*(*t*)), where *A* is the time‐varying amplitude and *q* is the time‐varying phase (see Figure 1C, left). We computed the dynamic phase‐locking matrix (dPL, with size N×N×T). Each entry of the dPL(*n*,*p*,*t*) estimates the phase alignment (which varies from −1 to 1) between brain regions *n* and *p* at time *t*, given by the following equation:

$$dPL\left(n,p,t\right)=cos\left(\theta \left(n,t\right)-\theta \left(p,t\right)\right),\;\mathrm{with}\;n,p\;=1,\text{…},N.$$

To characterize the evolution of the dPL matrix over time with reduced dimensionality, we considered only its leading eigenvector, *V_1_(t)*, which is a *Nx1* vector that captures, at time *t*, the projection of the BOLD phase in each brain area into the main *orientation* of BOLD phases over all areas (Figure 1C, second panel from the left). When all elements of *V_1_(t)* have the same sign, all BOLD phases project in the same direction with respect to the orientation determined by *V_1_(t)*. If instead the first eigenvector *V_1_(t)* has elements of different signs (i.e., positive and negative), the BOLD signals project into different directions with respect to the leading eigenvector, which naturally divides the brain into distinct modes (colored in red and blue in Figure 1C, second panel from the left). Previous studies using the LEiDA have shown that the subset of brain areas whose BOLD signals appear temporally phase‐shifted from the main BOLD signal orientation reveals meaningful functional brain networks [37, 39, 40, 41].

We chose to use the LEiDA because it allows for high temporal resolution and continuous tracking of brain state transitions without the need for fixed time windows, as in traditional windowed dynamic connectivity approaches. This method provides a more precise view of rapidly changing brain networks that are particularly relevant to the real‐time dynamics of musical improvisation.

### 2.9 Recurrent BOLD PL Substates

**FIGURE 2:**
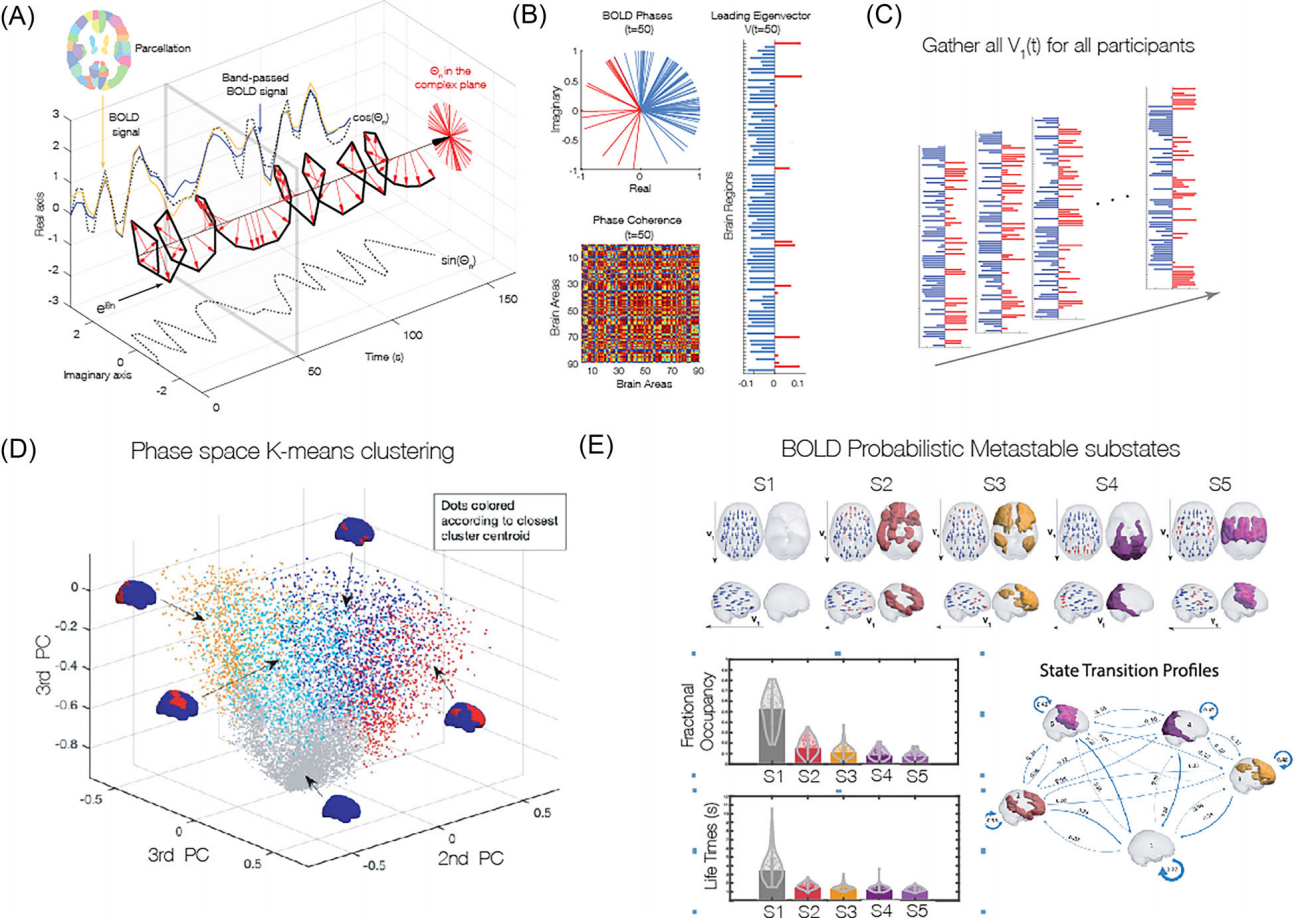
Fingerprinting the brain dynamics underlying improvisation. (A) The phase of the BOLD signal is computed over space and time, and a whole‐brain, phase‐coherence matrix represents the patterns of functional connectivity at each time point. (B) For all phase‐coherence matrices across participants and time points, the leading eigenvectors are extracted, setting the data foundation for characterizing the patterns of brain dynamics underlying a task or condition. (C) The Leading Eigenvector Dynamics Analysis (LEiDA) method characterizes the stochastic subdivisions of regular and persistent brain states, or the probabilistic metastable substates (PMS), from neuroimaging data. Here, a clustering algorithm determined the substates of brain activity, or PMS (i.e., the center of the cluster centroids), best representing the dynamics of the brain states. The resulting substates of brain activity are then typified by their probability of occurrence and lifetime, thus offering a statistically refined strategy for identifying the fundamental differences in neural dynamics underlying various conditions or brain states. Abbreviations: BOLD, blood oxygenation level‐dependent; PC, principal component.

In this work, we aimed to investigate the existence of specific patterns of BOLD PL substates (Figure 2), or PL substates, associated with musical creativity in jazz improvisation. To do so, we first searched for recurrent BOLD PL patterns emerging in each of the three experimental conditions (*byHeart*, *iMelody*, and *iFreely*), and compared their probabilities of occurrence to a common rest baseline. Recurrent PL substates were detected by applying a *k*‐means clustering algorithm to the set of leading eigenvectors, *V_1_(t)*, associated with the fMRI volumes acquired during each condition over all participants (considering a lag of three TR [3×1460 ms = 4380 ms] to account for the delay in the hemodynamic response), as well as the fMRI volumes recorded during a baseline (rest) period of 542 s (the same baseline was used for all three experimental conditions). The *k*‐means algorithm clusters the data into an optimal set of *k* clusters, where each cluster can be interpreted as a recurrent PL substate (Figure 2D).

While rest fMRI studies have revealed the existence of a reduced set of approximately 5−10 functional networks that recurrently and consistently emerge during rest across participants and recording sites [33, 37, 42, 43], the number of PL substates emerging in brain activity during a task is undetermined and depends on the level of precision allowed by the spatial and temporal scales of the recordings. In the current study, we did not aim to determine the optimal number of recurrent PL substates detected in a given condition, but instead to detect which clusters of the PL substates whose POc was significantly (Bonferroni correction adjusted *p*‐value < 0.05) and consistently modified by the experimental conditions with respect to the baseline. In that direction, we ran the *k*‐means algorithm for 13 partition models by varying the number of clusters *k* from 3 to 15, with higher *k* resulting in more fine‐grained configurations. Particularly, for each partition model, clustering the 23,744 leading eigenvectors (resulting from 16 participants and 1484 TRs each for the total recording time) into *k* clusters, returns *k* N X 1 cluster centroids, Vc, each representing a recurrent substate of BOLD PL. The brain substate related to each Vc can be represented as a network in a cortical space. Previous works have demonstrated that the smallest synchronized community in Vc has a highly significant overlap with functional networks [39, 44].

### 2.10 Probability of Occurrence

Recurrent substates were compared in terms of their POc (calculated as the number of time points in which a PL substate is active during the scan, divided by the total number of time points in a scan) in both strategies for improvisation (by *iMelody* and *iFreely*) and play from memory (*byHeart*) with respect to their probabilities of occurrence during the condition and rest baseline, using a permutation‐based paired *t*‐test to assess the statistical differences. The significant thresholds were corrected to account for multiple comparisons as 0.05/*k*, where *k* is the number of substates (or independent hypothesis) tested in each partition model [39, 44, 45].

### 2.11 Comparison With Rest Networks

We used the large‐scale rest networks (RSNs) described by Shirer and colleagues [46] to quantify the representation of each RSN in each of the five substates. The intersection of each of the 14 RSNs with the 90 AAL brain regions was computed. Quantification of each RSNs representation was then calculated by dividing the results of the intersection between RSNs and 90 AAL by the total number of voxels of each RSN intersected with the 90 AAL regions.

## 3 Results

In this study, we investigated the dynamic nature of the jazz musicians’ brains while they improvised on the melody and freely, by characterizing the most recurrent patterns of whole‐brain functional connectivity arising during the 6 min of playing in each condition. First, we analyzed participants’ improvisations using the behavioral MIDI data. Second, we analyzed the brain activity during improvisation.

### 3.1 Behavioral Results

#### 3.1.1 Number of Played Notes

**FIGURE 3:**
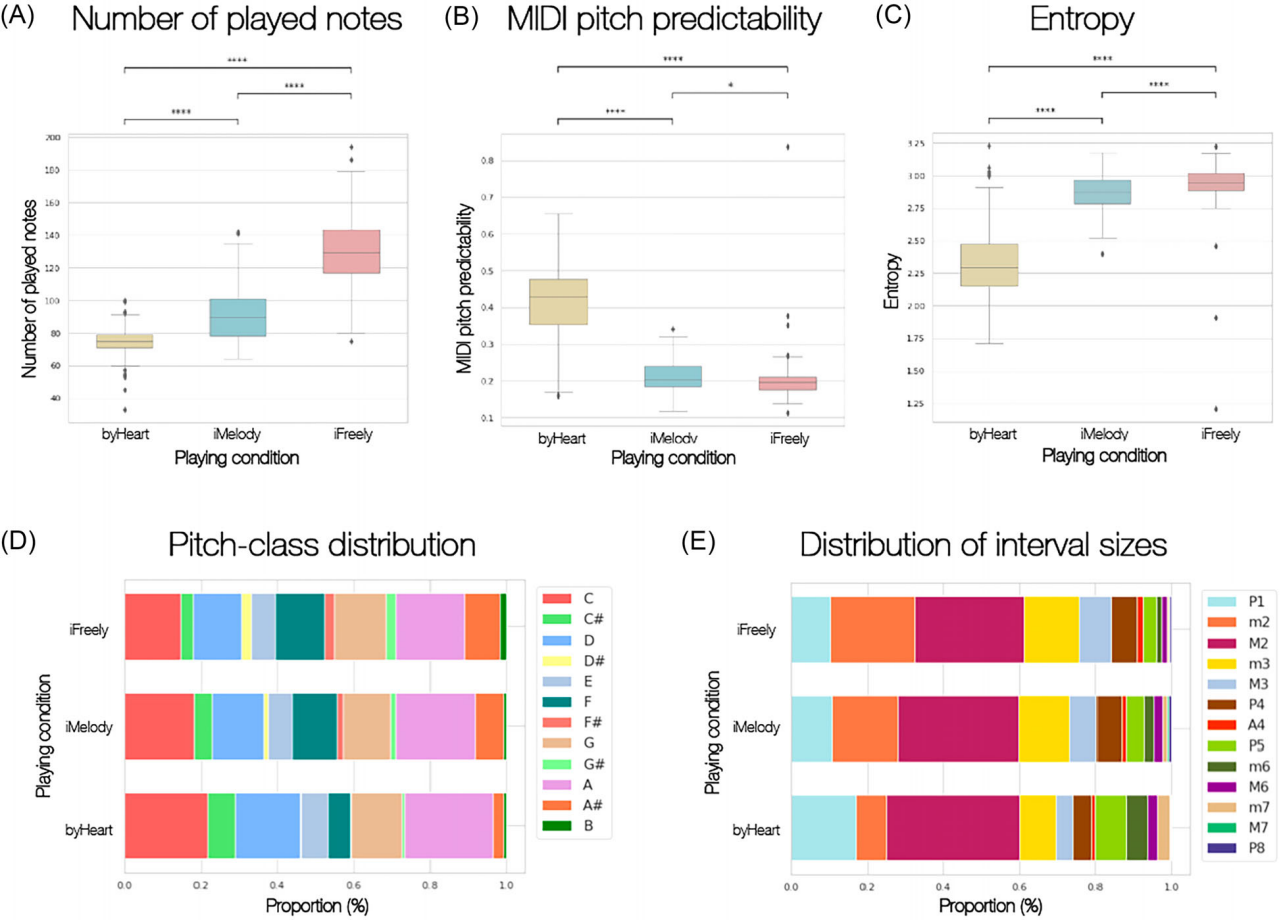
Music information retrieval across playing conditions. (A) Average number of played notes. Music improvisation is characterized by a significantly higher number of played notes (*p* < 0.05; Bonferroni corrected) when compared to *byHeart*, with *iFreely* surpassing *iMelody*, thus suggesting a clear positive association between the number of played notes and the level of improvisation. (B) Predictability in terms of MIDI pitch. Our results support the hypothesis that *iFreely* and *iMelody* are considerably less predictable than music played by heart (*byHeart*), with *iFreely* revealing the highest unpredictability level. (C) Entropy level. The *iFreely* and *iMelody* conditions are significantly more entropic than the *byHeart* condition, with free improvisation protruding as the most entropic playing strategy. (D) Distribution of pitch classes (PC), in percentage. The existence of a clear gradient effect in the distribution of PC across conditions, with *iMelody* setting the intermediate layer of metric complexity between *byHeart* and *iFreely*. (E) Distribution of interval classes (percentage). Over 90% of the played intervals corresponded to the perfect fourth (P4) or smaller intervals in *iFreely*, which is concomitant with the trend for musicians to progressively play more notes in *iFreely* than in *iMelody* and *byHeart*. Abbreviations: P1, perfect unison; m2, minor second; M2, major second; m3, minor third; M3, major third; P4, perfect fourth; A4, augmented fourth (tritone); P5, perfect fifth; m6, minor sixth; M6, major sixth; m7, minor seventh; M7, major seventh; P8, perfect octave.

Music improvisation is characterized by a significantly higher number of played notes compared to playing by heart (*p* < 0.05; Bonferroni corrected), with the highest count observed in the free improvisation condition (Figure 3A). This finding was as expected since the participants experienced fewer structural constraints while improvising, allowing for a more spontaneous note production.

#### 3.1.2 Pitch Predictability and Entropy

We observed significant differences (*p* < 0.05, Bonferroni corrected) in MIDI pitch predictability across conditions (Figure 3B,C). Improvisation modes exhibited significantly lower predictability and higher entropy compared to the *byHeart* condition, with *iFreely* displaying the greatest level of unpredictability. These findings suggest that improvisation, particularly in its most unconstrained form, fosters a greater degree of musical novelty and complexity.

#### 3.1.3 Pitch Classes

The distribution of pitch classes (PC) across conditions (Figure 3D) predominantly followed the F major scale notes, which corresponds to the original key of the performed jazz standard. As expected, this tendency was most pronounced in the *byHeart* condition, where musicians relied on memorized patterns and aimed to precisely replicate the original melodic line. Our results suggest the existence of a gradient effect on PC distribution across conditions, with lower‐ and higher‐range PC occurring more frequently in the improvisation modes (highest occurrence in *iFreely*). *iMelody* formed an intermediate layer of metric complexity. These results are consistent with our hypothesis that the degree of metric complexity constitutes an important predictor of musical creativity.

#### 3.1.4 Distribution of Interval Sizes

With respect to interval distribution, over 90% of the intervals played in *iFreely* corresponded to perfect fourths (P4) or smaller intervals, reinforcing the trend that musicians tend to play more notes in *iFreely* compared to *iMelody* and *byHeart*. This increase in note density is accompanied by a preference for smaller intervals (Figure 3E). Notably, a significant proportion of these intervals involve chromatic alterations outside the diatonic scale, evidenced by the increased presence of D#, F#, and G#. This suggests that, despite the underlying harmonic structure provided by the chord changes of the backing track, the greater creative freedom in *iFreely* allows musicians to explore a wider harmonic palette, incorporating more nondiatonic elements into their improvisation.

### 3.2 Detection of the PL Substates

The repertoire of metastable substates depends upon the number of clusters determined by the k‐means clustering algorithm, where a higher number of clusters usually results in less frequent and more fine‐grained substates [45]. In this study, we searched for the PL substates that significantly and recurrently characterize musical improvisation using a memory condition and rest as a baseline.

**FIGURE 4:**
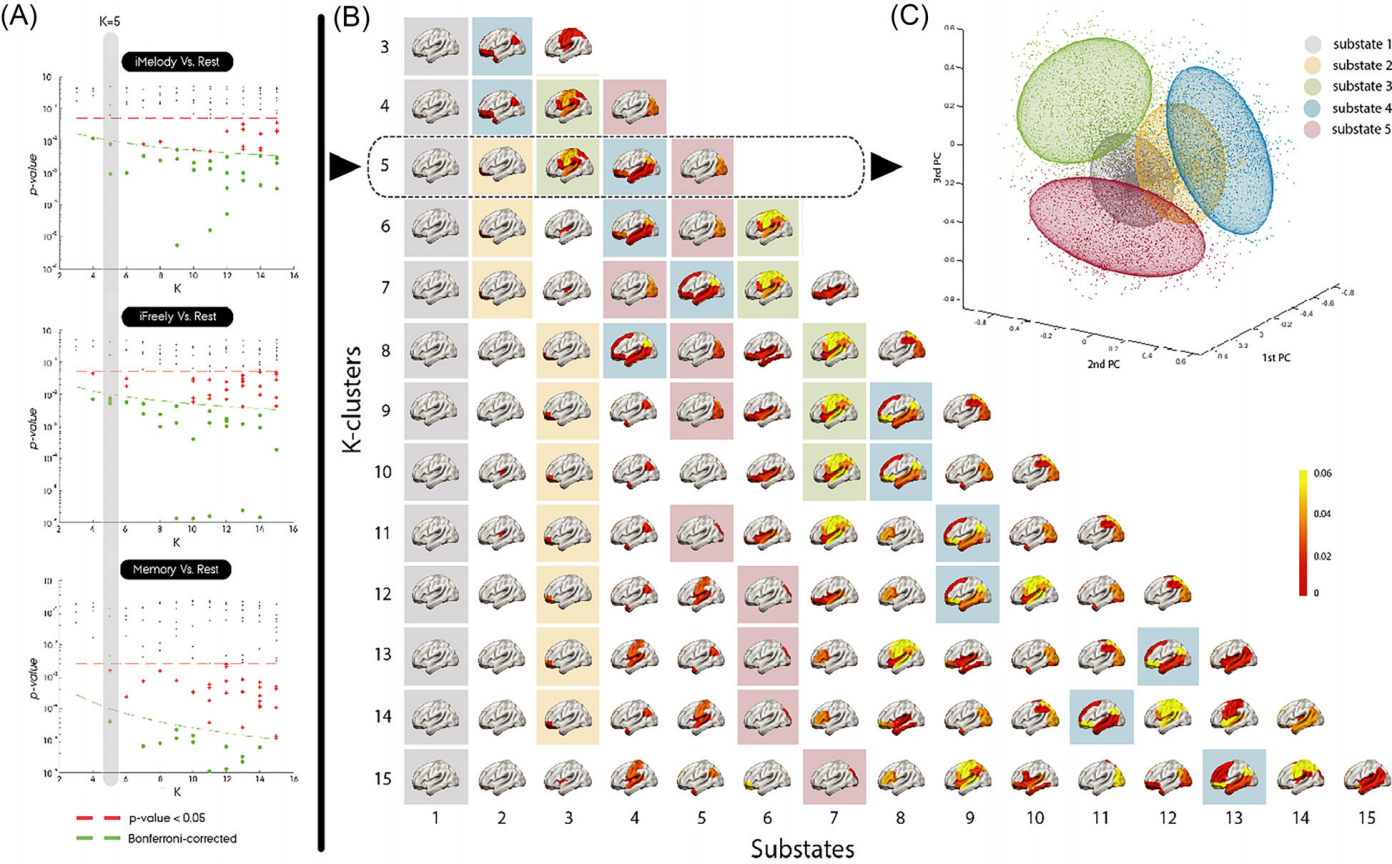
Detection of optimum number of substates. (A) For each pair of conditions and partition model (k‐means clustering solution), we plot the *p*‐values associated with the between‐condition comparison between experimental condition and rest in terms of probability. The *p*‐values marked as green dots survive the correction for multiple comparisons within each partition model (<0.05/*k*). The black dashed line highlights the partition model chosen (*k* = 5; the lowest *k*‐value with significant statistical difference between each of the music playing conditions and the rest condition) to describe the clustered dynamics of the experimental conditions under study. (B) Brain rendering of the phase‐locked (PL) substates across partition models. Brain regions are color‐coded according to PL amplitude of the eigenvector representing them. Here, we used the Dice Similarity Coefficient (DSC) to highlight (background squared shade) how consistent the substates are across *k*‐solutions, with regard to *k* = 5. Using the reference substate color‐coding of *k* = 5, across clustering solutions, substates are highlighted with the corresponding color of the reference substate if it shares a DSC ≥ 0.8. (C) The BOLD PL patterns (leading eigenvectors of BOLD phases) captured for all time points, conditions, and subjects can be represented in a three‐dimensional version of the phase space. Here, each data point (dot) is placed according to their cosine distance to the three principal components, or eigenvectors of the covariance matrix, estimated from all observations. Observations are colored according to the cluster they are assigned to for *k* = 5 (i.e., the closest cluster centroid). Color‐coding is congruent with the brain renderings projecting each of the cluster centroids (i.e., each PL substate). Additionally, ellipsoids are fitted to each set of data points to represent the degree of dispersion and directionality of each cluster cloud. Abbreviations: BOLD, blood oxygenation level‐dependent; PC, principal component.

We first estimated the PL substates that most significantly and consistently differentiated improvisation during *iMelody* and *iFreely* from the baseline condition (rest). Across all 13 partition models produced, a total of 12 PL substates were found to be significantly different (Bonferroni corrected) between improvisation strategies and rest in terms of POc. For each partition model, statistical significance between pairs of conditions (in terms of probability and duration of the PL substates) was assessed using permutation‐based paired *t*‐tests between‐condition comparison (Figure 4, left), and corrected for multiple comparisons using Bonferroni's method. Based on the criteria for the minimum number of PL substates revealing significant differences in POc between all music‐playing strategies under study and the baseline conditions, we selected the partition solution that divides the brain dynamics of all conditions into five PL substates (*k* = 5, stippled line in Figure 4). The BOLD PL patterns captured for all time points, conditions, and subjects are clustered into five cluster centroids. The partition into five substates is in line with the literature, where 5−10 functional networks emerge during rest [37, 42]. Furthermore, as highlighted in Figure 4 (left), all five substates derived at the chosen *k*‐level consistently reappeared across multiple partition solutions (Dice Similarity Coefficient ≥ 0.8; *k* ∈ [5, . ., 9]), thus strengthening the reliability of this method and criteria used in capturing a robust signature of brain dynamics across all conditions under study.

### 3.3 Repertoire of Recurrent PL Substates

In line with previous studies using LEiDA, the most probable state of BOLD PL is a global substate, where all BOLD signals are synchronized. The remaining four recurrent substates were found to overlap with typical RSNs reported in the literature [46].

### 3.4 POc

**FIGURE 5:**
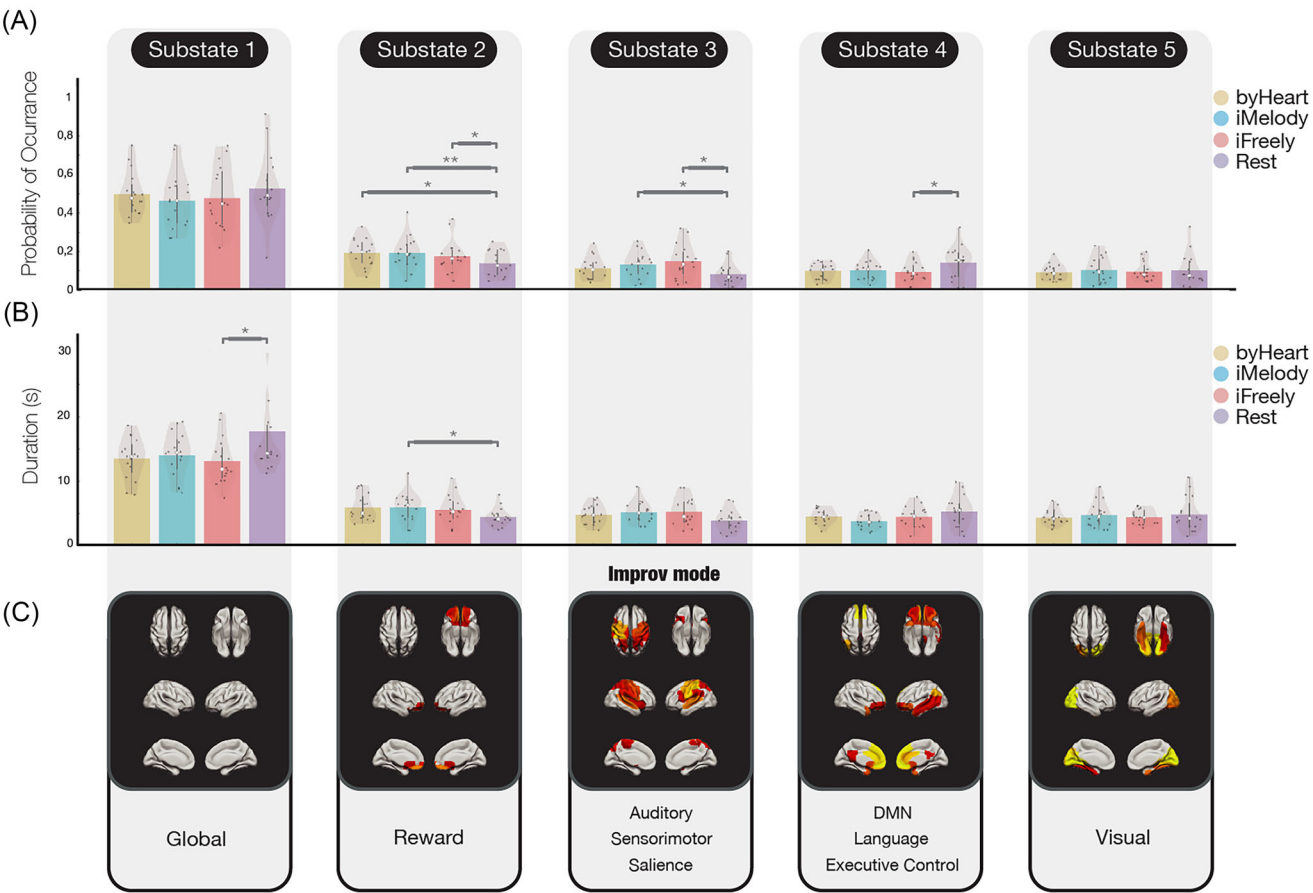
Brain dynamics underlying musical improvisation. Characterizing the repertoire of metastable substates during jazz improvisation (*iMelody* and *iFreely*), play from memory (*byHeart*), and rest. (A) Probability of occurrence (POc) of each of the five brain substates estimated using The Leading Eigenvector Dynamics Analysis (LEiDA), during play from memory (yellow), improvisation on the melody (cyan), improvisation freely (red), and rest (purple), represented by bar and violin plots. Substate 3 was found to have significantly (*p*<0.05; Bonferroni‐corrected) higher POc in both strategies for improvisation (*iMelody* and *iFreely*) than in rest and substate 4 had significantly lower POc in *iFreely* than in rest. (B) Duration of each of the five brain substates. (C) Rendering of the five substates and corresponding rest networks (shown from top, bottom, and side [hemispheric] planes). The results revealed five recurrent phase‐locked substates, one global substate (substate 1), and four recurrent substates, reflecting: reward (substate 2); an auditory–motor network (substate 3); a complex array of functions that support improvisation and creativity more generally (such as evaluation‐perception) (substate 4); and visual imagery (substate 5).

When compared with rest, performing in any of the three conditions (*byHeart*, *iMelody and iFreely*) revealed a reward‐related network, substate 2, which includes the superior, medial, and middle orbitofrontal and olfactory cortices bilaterally, and shows a significantly higher POc during the musical tasks compared to rest (Figure 5).

We found a recurrent substate, substate 3, with significantly higher POc for both strategies for improvisation (*iMelody* and *iFreely*) compared to rest (Figure 5). This PL substate includes the bilateral: precentral gyrus, Rolandic operculum, posterior cingulate gyrus, superior parietal gyrus, supramarginal gyrus, Heschl's gyrus, superior temporal gyrus; the left: IFG opercularis, paracentral lobule, and pallidum; and the right insula. These brain regions are mainly part of the auditory, sensorimotor, and posterior SNs.

Interestingly, another recurrent substate, substate 4, was found to have a lower POc (but with higher duration) in *iFreely* than in *iMelody*, and significantly lower POc in *iFreely* than at rest (Figure 5). This PL substate includes the bilateral: orbital frontal cortex (inferior, middle, and superior), olfactory cortex, medial prefrontal cortex, ventromedial prefrontal cortex, medial orbitofrontal cortex, anterior cingulum, and middle temporal gyrus; the left: posterior cingulum, angular gyrus, and superior temporal gyrus. These brain regions are mostly part of the DMN, and the language and left ECN networks.

## 4 Discussion

In the present study, we investigated the brain activity in jazz musicians playing the jazz standard DWR while systematically increasing the level of improvisational freedom: from playing a melody by heart (*byHeart*) over improvising on either the melody (*iMelody*) or freely on the harmonic progression (*iFreely*). We found a significantly higher POc of brain networks in a brain substate (*improv mode*) predominantly comprising the auditory, sensorimotor, and posterior SNs when contrasting *iMelody* and *iFreely* to rest. In addition, we found that the highest level of improvisational freedom (*iFreely*) was characterized by a lower occurrence of another specific brain substate comprising mainly areas of the dorsal DMN, the left ECN, and the anterior SN with a significantly higher POc in rest than in *iFreely* (a more free strategy for improvisation), with *iFreely* having the lowest POc of all the four conditions.

Behaviorally, the increase in improvisational freedom corresponded to an increasing number of notes played by the musicians, as well as higher levels of unpredictability and entropy. Previous studies (e.g., Ref. 7) have linked DMN engagement to creative cognition; our findings suggest that in the highly active and sensorimotor‐driven state of *iFreely*, creativity may be supported by a different configuration of brain networks. This could reflect the real‐time demands of motor execution and auditory feedback processing in spontaneous performance, which might downregulate internally oriented processes typically associated with the DMN.

These behavioral results can be seen in the light of important models of music improvisation, such as Loui's influential multilevel model of musical creativity [4]. In this model, musical improvisation is a complex system embodying a knowledge base that is expanded by predictions over different timescales [4]. The model emphasizes that musicians use an existing knowledge base and a vocabulary shared between participating musicians as a prerequisite for improvisational interaction.

The increasing entropy and unpredictability related to the *improv modes* indicates that musicians here use a larger vocabulary in a less predictable way than when playing *byHeart*. It is important to note, however, that in a more modern jazz repertoire (e.g., bebop compositions) rather than the jazz standard used in the present study, there is higher entropy and greater unpredictability [47]. The idea of jazz improvisation as manipulations of a certain musical vocabulary resonates well with earlier findings emphasizing similarities to spoken language [15, 48]. When musicians improvise, they exchange phrases and cue certain replies from the other musicians, and this interaction can be shown to mimic the functions found in language communication [12].

The behavioral results are supported by the brain data, which showed that the repertoire of brain dynamics increased with increasing levels of improvisational freedom. Below, we discuss our findings in terms of the differences in dynamic structure by characterizing the brain substates that best describe the brain dynamics of *byHeart*, *iMelody*, *iFreely*, and rest in jazz musicians, and the POc of these substates.

Our results revealed an optimum of five substates, which is in line with previous studies using LEiDA [37, 39, 44, 45]. Figure 5 shows a global substate (substate 1) and four recurrent substates that overlap with known RSNs reported in the literature [43, 46]. These four recurrent substates reflect: reward (substate 2), *improv mode* comprising an auditory–motor network (substate 3), a complex array of functions that support music creation “on the fly,” involving intuitive decision‐making (evaluation/perception) (substate 4), and a visual network (substate 5). As such, these five brain substates reflect the model of improvisation proposed by Pressing, where improvisation is described to be a dynamic interplay between motivation, generation, evaluation, and execution of novel motor sequences [3].

Playing music in general (*byHeart*, *iMelody*, and *iFreely*) resulted in a higher POc of substate 2 (*reward mode)*, compared to rest. This was expected given the well‐known association between music and activity in reward‐related networks [49, 50, 51]. This substate comprised emotional regions such as the bilateral superior, medial, and middle orbitofrontal and olfactory cortices. These regions cluster bilaterally around the orbitofrontal cortex, a region known to be a nexus for sensory integration, prediction‐monitoring, and reward [52]. While some earlier studies have noted the significance of emotional regions in musical improvisation [4, 53], other studies of musical creativity do not report activity in these regions. In these studies, however, researchers use another playing condition to contrast with an improvisation task in music creativity studies [19, 53], whereas in the present study, we contrasted the playing conditions with a rest. It is also well‐known that many early fMRI studies suffer from signal dropout in the orbitofrontal regions.

Hence, substate 2 occurred in all playing conditions regardless of the level of improvisational freedom and could be an expression of the subjective enjoyment of playing music in general. In fact, even though substate 2 had a significantly higher POc in all playing conditions compared to the rest, *byHeart* had the highest POc. Music is known to be as rewarding as food or sex [54], and is rated to be on the top 10 things that people find most pleasurable in life [55]. This opens up to future questions as to whether reward, pleasure, and the drive to create are linked and essential for the creative process to happen, or if it is a domain‐specific process of musical activities.

Specific to improvisation, substate 3 (*improv mode*) showed a significantly higher POc for both strategies for improvisation (*iMelody* and *iFreely*) when compared to rest. The *improv modes* comprised brain regions including the bilateral: rolandic operculum, pre‐ and post‐central gyrus, superior parietal gyrus, supramarginal gyrus, Heschl's gyrus, and superior temporal gyrus; the left: paracentral lobule, pallidum, and IFG opercularis; and the right insula. This substate covers mainly areas of the auditory, sensorimotor, and posterior SNs.

Sensorimotor regions have been reported to be involved in artistic creativity studies. A recent meta‐analysis in artistic creativity (music, literary, and drawing) reported common domain‐general brain activity patterns in the presupplementary motor area, left dorsolateral prefrontal cortex, and right IFG across the different types of creativity [56]. However, the authors also found domain‐specific musical creativity activity in the left: supplementary motor area, precentral gyrus, middle frontal gyrus, and bilateral IFG. Increased connectivity of sensorimotor regions has been linked to higher levels of expertise in improvisation when comparing musicians with different levels of expertise in improvisation [21].

It is known that sensorimotor networks play an important role in music performance. Often, jazz musicians report that they “hear” (auditory imagery) what they intend to play even before playing it [57]. Hence, they form predictions about what the music will sound like even before the actual performance. These auditory–motor networks have, however, not always been observed in previous studies of musical improvisation compared to control tasks [58]. In a review of 16 musical creativity studies, Bashwiner and Bacon [59] reported that improvisation was associated with more activity in motor regions, but only half of the studies reported the same for auditory‐associative regions, such as in the present study.

Importantly, in the present study, substate 4 showed a lower POc for free improvisation but only compared to rest. Here, *iFreely* involves soloing on the given chord scheme without the limitations of having to create musical phrases resembling the melody of the jazz standard. This allowed the participants to use a larger repertoire of melodic and rhythmic material, as shown by the behavioral data. In this study, the *iFreely* condition corresponds closely to the unconstrained improvisation performed by jazz musicians in a natural playing situation, whereas the *iMelody* leads to a more constrained improvisation.

Substate 4 comprises brain regions of the dorsal DMN, language networks, and the left ECN, including the bilateral: ventromedial prefrontal cortex, medial prefrontal cortex, all sections of the orbital frontal cortex, olfactory cortex, middle temporal poles, anterior cingulum; and the left: angular gyrus, posterior cingulum, and the middle temporal gyrus. This is consistent with previous research where the DMN (responsible for spontaneous and self‐generated thought) and the ECN (responsible for cognitive control and maintenance of goal‐directed cognitive processes) are shown to cooperate in order to generate and evaluate ideas during creative tasks [7, 19, 21].

One may speculate that substate 4 involves an interaction between different cognitive processes such as idea generation (where attention, memory retrieval, and mind‐wandering are needed), selection, evaluation (of idea and reward value), and perception. In music improvisation, this lower predominance may be explained by the fact that in order to overcome knowledge constraints, a downregulation of prefrontal cortex regions (i.e., reduced cognitive control) and lower recurrence to salient information is needed [60, 61]. Lesion studies in the dorsolateral prefrontal cortex and orbitofrontal cortex have been related to higher performance on problem‐solving tasks and an increased capacity to overcome salient information during the idea generation stage [61]. Of note, this substate was found to have a slightly higher dominance in *iMelody* than in *iFreely*, which suggests that different improvisational strategies may rely upon different cognitive processes.

Overall, our study sheds new light on the brain dynamics of jazz improvisation. Earlier studies on jazz improvisation have consistently implicated two types of networks: domain‐specific networks related to movements, motor sequence generation, sensorimotor integration, and auditory cortices [14, 19]; and domain‐general networks involved in attention, executive control, emotional processing, and interpersonal communication [4, 17, 18]. This is consistent with the central components of formal musical training, as typically emphasized in music schools and conservatories. In particular, three domain‐general networks have been suggested to be crucial for improvisation: (1) DMN [62] related to idea generation and spontaneous thought; (2) the SN [63], important for evaluating ideas and the integration of emotional and cognitive resources; and (3) the ECN [64] responsible for goal‐oriented decision‐making.

In the present study, these three networks were integrated in substates 3 and 4, albeit in different ways. Substate 4 includes the DMN and the ECN and occurred less in *iFreely* than in rest. This is in contrast to substate 3, which included the SN and occurred more in both improvisation strategies compared to *byHeart* and rest.

An important consideration is what insights the present study offers to the nature of creativity more broadly. Some researchers define creativity as the ability to produce outcomes that are both novel and useful. With regard to novelty, the present behavioral results with less predictability and higher entropy in improvisation indicate that higher degrees of improvisational freedom are indeed more novel than playing the theme by heart. With regard to the usefulness of music, this is harder to define and assess. Future studies could rate the generated music for pleasure or liking as a proxy for usefulness. Importantly, however, there is a nonlinear relationship between complexity and rated pleasure, which typically follows an inverted U‐shaped curve (the so‐called Wundt‐curve) [65, 66], where medium complexity is rated higher than both low and high complexity. So, even though the *iFreely* condition may result in the most novel musical expression, it may not be the highest rated in terms of pleasure or liking, and hence not be seen as useful.

## 5 Conclusion

In sum, this study offers novel insights into the brain dynamics underpinning musical improvisation by identifying specific substates associated with different levels of creative freedom. By integrating dynamic functional connectivity analysis with a graded improvisation paradigm, we showed that increasing improvisation freedom corresponds to a shift in the balance of brain network engagement—from greater involvement of executive and evaluative networks (substate 4) in constrained improvisation to heightened activity in auditory–motor and SNs (substate 3) during more free‐form creativity. These findings expand upon previous models of improvisation by emphasizing the dynamic reconfiguration of domain‐specific and domain‐general networks, and highlight the importance of network interplay over time rather than static activation alone. This approach refines existing frameworks of musical creativity and offers a scalable approach for probing the neural basis of spontaneous creative behavior.

## Author Contributions

P.A.D.M., H.M.F., M.L.M.L.K., and P.V. conceived the experiment, hypotheses, and designed the study. M.L.M.L.K. and P.V. recruited the resources for the experiment. P.A.D.M., H.M.F., and O.A.H. collected the data. P.A.D.M., H.M.F., J.V., and A.T.L.Q. analyzed the data and performed statistical analysis with the contribution of E.S.E., J.C., O.A.H., N.S., and G.D. M.L.M.L.K. and P.V. provided guidance to interpret and frame the results within the behavioral and neuroscientific literature. P.A.D.M., H.M.F., A.T.L.Q., M.L.M.L.K., and P.V. wrote the first draft of the manuscript. P.A.D.M., H.M.F., A.T.L.Q., and M.L.M.L.K. prepared the figures. All authors contributed to and approved the final version of the manuscript.

## Conflicts of Interest

The authors declare no competing interests.

## Data Availability

The data that support the findings of this study are available on request from the corresponding author. The data are not publicly available due to privacy or ethical restrictions.
